# How to Determine the Accuracy of an Alternative Diagnostic Test when It Is Actually Better than the Reference Tests: A Re-Evaluation of Diagnostic Tests for Scrub Typhus Using Bayesian LCMs

**DOI:** 10.1371/journal.pone.0114930

**Published:** 2015-05-29

**Authors:** Cherry Lim, Daniel H. Paris, Stuart D. Blacksell, Achara Laongnualpanich, Pacharee Kantipong, Wirongrong Chierakul, Vanaporn Wuthiekanun, Nicholas P. J. Day, Ben S. Cooper, Direk Limmathurotsakul

**Affiliations:** 1 Mahidol-Oxford Tropical Medicine Research Unit, Faculty of Tropical Medicine, Mahidol University, Bangkok, Thailand; 2 Centre for Tropical Medicine and Global Health, Nuffield Department of Clinical Medicine, University of Oxford, Oxford, United Kingdom; 3 Chiang Rai Prachanukroh Hospital, Chiang Rai, Thailand; 4 Department of Tropical Hygiene, Faculty of Tropical Medicine, Mahidol University, Bangkok, Thailand; University of Texas Medical Branch, UNITED STATES

## Abstract

**Background:**

The indirect immunofluorescence assay (IFA) is considered a reference test for scrub typhus. Recently, the Scrub Typhus Infection Criteria (STIC; a combination of culture, PCR assays and IFA IgM) were proposed as a reference standard for evaluating alternative diagnostic tests. Here, we use Bayesian latent class models (LCMs) to estimate the true accuracy of each diagnostic test, and of STIC, for diagnosing scrub typhus.

**Methods/Principal Findings:**

Data from 161 patients with undifferentiated fever were re-evaluated using Bayesian LCMs. Every patient was evaluated for the presence of an eschar, and tested with blood culture for *Orientia tsutsugamushi*, three different PCR assays, IFA IgM, and the Panbio IgM immunochromatographic test (ICT). True sensitivity and specificity of culture (24.4% and 100%), 56kDa PCR assay (56.8% and 98.4%), 47kDa PCR assay (63.2% and 96.1%), *groEL* PCR assay (71.4% and 93.0%), IFA IgM (70.0% and 83.8%), PanBio IgM ICT (72.8% and 96.8%), presence of eschar (42.7% and 98.9%) and STIC (90.5% and 82.5%) estimated by Bayesian LCM were considerably different from those obtained when using STIC as a reference standard. The IgM ICT had comparable sensitivity and significantly higher specificity compared to IFA (p=0.34 and p<0.001, respectively).

**Conclusions:**

The low specificity of STIC was caused by the low specificity of IFA IgM. Neither STIC nor IFA IgM can be used as reference standards against which to evaluate alternative diagnostic tests. Further evaluation of new diagnostic tests should be done with a carefully selected set of diagnostic tests and appropriate statistical models.

## Introduction

Scrub typhus, a bacterial infection caused by *Orientia tsutsugamushi*, is endemic in South, East and Southeast Asia, as well as some areas in northern Australia [1,2]. The organism is transmitted to humans through skin inoculation by larval *Leptotrombidium* mites (chiggers). Scrub typhus can be severe and fatal when left untreated, with reported mortality ranging from 14% to 30% in Southeast Asia [3–5]. The diagnosis of scrub typhus is difficult. Patients with scrub typhus often come to hospital with undifferentiated fever and symptoms that are similar to other endemic infections such as leptospirosis, malaria and dengue. An eschar, a necrotic lesion formed at the site of inoculation, is the most characteristic sign of scrub typhus. However, an eschar is not observed in every scrub typhus patient, and similar lesions can also be observed in patients with other diseases such as spider bites, spotted fever group rickettsioses, and cutaneous lesions caused by tuberculosis, leishmaniasis and anthrax [6,7].

There are two main laboratory methods for diagnosing scrub typhus, namely bacterial and antibody detection. Bacterial detection methods include isolation of *O*. *tsutsugamushi* (culture) and polymerase chain reaction (PCR) assays targeting the 56kDa, 47kDa, and *groEL O*. *tsutsugamushi* genes [8–12]. Antibody detection methods include the indirect immunofluorescence antibody assay (IFA), the indirect immunoperoxidase assay (IIP), the Weil-Felix test, and various commercially available immunochromatographic tests (ICT) [8,13–15]. IFA uses fluorescent anti-human antibody to detect the presence of antibody specific to *O*. *tsutsugamushi* in patient serum, and is regularly used as a reference test against which alternative diagnostic tests for scrub typhus are evaluated [8,16]. However, IFA has several limitations [16]. The cut-off antibody titre of IFA for acute serum samples remains controversial, the determination of IFA results is subjective, and the true accuracy of IFA is suspected to be imperfect [8].

We recently proposed the Scrub Typhus Infection Criteria (STIC), a combination of culture, PCR assays, and IFA IgM, as a reference standard for scrub typhus diagnosis [17,18]. STIC is considered positive if either (a) *O*. *tsutsugamushi* is isolated, (b) at least two out of three PCR assays targeting the 56kDa, 47kDa and *groEL* genes are positive, (c) an admission IFA IgM titre is ≥ 1:12,800 or (d) there is at least a four-fold rise in convalescence IFA IgM titre compared to the admission IFA IgM titre [17,18]. The development and details of STIC, including selection of the cut-off titres of IFA IgM for STIC, are described elsewhere [17,18]. In short, STIC were designed based on available diagnostic tests to provide a robust set of criteria for a final diagnosis of acute scrub typhus infection with a high level of confidence in a research setting, and STIC have already been used as a comparator to evaluate the accuracy of several alternative diagnostic tests [17,18].

Nonetheless, we hypothesized that STIC, when used as a comparator, might have been falsely assumed to be perfect (100% sensitivity and 100% specificity), and as a consequence the accuracy of the alternative diagnostic tests might have been inaccurately estimated. Bayesian latent class models (LCM) are increasingly used to estimate accuracy of diagnostic tests since it does not need to assume that the accuracy of reference tests is perfect [19–24]. In this study, we re-analyzed our existing data set from a previously published prospective study [17,18] and estimated the true accuracy of each diagnostic test for scrub typhus using Bayesian LCM.

## Materials and Methods

### Study patients

The data set analysed in this study was from a prospective study of acute febrile illness in a scrub typhus endemic area, Chiang Rai, Northern Thailand [17,18]. In brief, a total of 161 patients were recruited into the study from August 2007 to August 2008 [17,18]. All participants were over 15 years old, presented with acute febrile illness for less than 2 weeks, had three negative malaria blood smears, and had no evidence of a primary focus of infection. Every patient was examined for the presence of an eschar. Admission and convalescent blood samples were collected for a set of diagnostic tests [17,18].

### Ethical statement

Ethical approval for the prospective study was obtained from the ethical committees of Chiang Rai Hospital, the Ministry of Public Health, Thailand, and from the Oxford Tropical Research Ethics Committee, United Kingdom. Signed written inform consent was obtained from every subject enrolled into the study [17,18].

### Diagnostic tests

All patient specimens were tested for evidence of acute scrub typhus infection using a set of bacterial and antibody detection methods [17,18]. All of the tests were done and reported in the previous publications [17,18]. In short, the bacterial detection tests were performed on admission samples and included *in vitro* isolation of *O*. *tsutsugamushi* (culture), nested 56kDa PCR assay, 47kDa-based real-time PCR assay, and *GroEL*-based real-time PCR assay as described previously [12,17,25–27]. Antibody detection tests performed included on-admission ICT (PanBio ICT, Australia) and paired IFA, both targeting specific IgM antibodies against *O*. *tsutsugamushi*.

IgM-based IFA was used to measure IgM-specific titers against pooled whole-cell antigens from three strains of *O*. *tsutsugamushi* (the Karp, Kato and Gilliam type strains) as previously described [16]. The scrub typhus IgM ICT was a prototype rapid diagnostic test developed by Panbio Company, Australia, and was performed according to the manufacturer’s instructions. The antigen used in the Panbio IgM ICT was a truncated recombinant 56-kDa protein of *O*. *tsutsugamushi* Karp strain [28].

### Statistical analysis

#### STIC as gold standard in Gold Standard Model

The accuracy of each diagnosis test was estimated by comparing with STIC, which were considered as a perfect reference test (100% sensitivity and 100% specificity), as previously described [17,18]. STIC was considered positive if either (a) *O*. *tsutsugamushi* was isolated, (b) at least two out of three PCR assays targeting the 56kDa, 47kDa and *groEL* genes were positive, (c) an admission IFA IgM titre was ≥ 1:12,800 or (d) there was at least a four-fold rise in convalescence IFA IgM titre compared to the admission IFA IgM titre [17,18]. A combination of two diagnostic tests was considered positive if either one of those tests was positive. Prevalence, sensitivity, specificity, positive, and negative predictive values (PPV and NPV) of the tests with their respective 95% confidence intervals (CI) were calculated using STATA 11.1 (Stata Corp., College Station, Tex.). The results obtained were comparable to those previously published [17,18].

#### Bayesian Latent Class Models

The true accuracy of each diagnostic test was then estimated using Bayesian LCMs. Detailed explanation on how Bayesian LCMs determine the unbiased accuracy of diagnostic tests has been given elsewhere [20–24,29,30]. In brief, Bayesian LCMs estimate accuracies of diagnostic tests based on the true disease status of each patient (infected or non-infected). Bayesian LCMs do not assume that any diagnostic test or combination of diagnostic tests is perfect. Diagnostic tests in the model included culture, a combination of PCR assays, paired IFA IgM, PanBio IgM ICT and presence of eschar. The combination of PCR assays and paired IFA IgM was defined according to the definition of STIC, which considered PCR assays positive when at least two out of three PCR assays, targeting 56kDa, 47kDa and *groEL* genes, were positive, and IFA IgM positive when admission IFA IgM titre was ≥ 1:12,800 or there was at least a four-fold rise in convalescence IFA IgM titre compared to admission IFA IgM titre.

To estimate the accuracy of a diagnostic test using LCMs, the best-fitting model, as determined by the presence or absence of correlation between diagnostic tests, was used. We evaluated possible correlations based on existing knowledge and external evidence. Therefore, correlations between bacterial detection tests (culture and a combination of PCR assays) and between antibody detection tests (IFA IgM and PanBio IgM ICT) were evaluated. As the Deviance Information Criterion (DIC) is not applicable to the random effect model used, the Akaike Information Criterion (AIC) was used to evaluate goodness of fit and to compare the models. A difference in AIC of more than 10 was taken as indicating definite support for the model with the lower value, while a difference between 5 and 10 was considered substantial support, and less than 5 inconclusive support.

The best-fitting model was used to determine the accuracy of each diagnostic test evaluated and combinations thereof. The Bayesian p values were used to compare accuracies between diagnostic tests. All Bayesian LCMs assumed that no prior information (non-informative priors) about unknown parameters (i.e. prevalence, sensitivities and specificities) was available, except that the specificity of culture was fixed at 100%. Sensitivity analysis was performed in which different prior information were used. All parameters with respective 95% credible intervals (CrI) were estimated using a Markov chain Monte Carlo method and WinBUGS 1.4 [31]. Interquartile ranges (IQRs) are presented as 25^th^ and 75^th^ percentiles. S1 and S2 Text provide full data sets and all of the models used, respectively. S1 and S2 Tables provides details about the method and the results for the best-fitting model selection.

#### Post-hoc model evaluation

We searched for other complete individual-level data sets in PubMed using MeSH terms (“Scrub Typhus” and “Sensitivity and Specificity”) for analysis with Bayesian LCMs. To validate the low sensitivity of IFA estimated by the Bayesian LCMs, we also estimated naïve sensitivities of those tests in only those who were culture and/or PCR assay positive in our data set [23]. To validate the low specificity of IFA estimated by the Bayesian LCMs, we evaluated the IFA results in patients with a firm diagnosis of other diseases in the data set, as previously described [18]. Murine typhus was diagnosed by IFA detecting antibody to the *Rickettsia typhi* Wilmington strain in paired specimens using slides purchased from the Australian Rickettsial Reference Laboratory[18]. Dengue was diagnosed using Panbio ELISA tests (Panbio, Brisbane Australia) to detect NS1 antigen and IgM antibodies in paired specimens [18].

## Results

One hundred and sixty one patients with acute febrile illness were recruited into the study. The median age was 41 years (IQR 29–51, range 15–81), and 99/158 (63%) were male. The median fever days before admission to hospital was 5 days (IQR 3–7 days), and convalescent samples were available for 138 patients (85.7%). Fifty-five were diagnosed with acute scrub typhus infection by STIC, giving a prevalence of 34.2% (95%CI 26.9–42.0%). There were 9, 27, 46 and 31 patients with positive scrub typhus test results on culture, using a combination of PCR assays, with the IFA IgM and with the PanBio ICT IgM, respectively. Seventeen patients had an eschar.

### STIC as gold standard

Table 1 shows the accuracy of each diagnostic test estimated by comparing it with STIC and assuming that STIC are a perfect reference. The sensitivity of IFA IgM was estimated to be 83.6%, and its specificity was assumed to be perfect (100%).

**Table 1:** Prevalence of scrub typhus and accuracy of diagnostic tests, estimated using the Scrub Typhus Infection Criteria (STIC) as the gold standard or using Bayesian latent class models (LCMs).

Parameters	STIC as the gold standard (95% CI)^†^	Final Bayesian LCM (95% CrI)^††^
**Prevalence**	34.2 (26.9–42.0)	23.0 (15.9–31.5)
**STIC**		
Sensitivity	100	90.5 (79.6–100)
Specificity	100	82.5 (79.4–85.6)
PPV	100	60.0 (51.0–69.1)
NPV	100	97.2 (91.5–100)
**Blood culture for *O*. *tsutsugamushi***		
Sensitivity	16.4 (7.8–28.8)	24.4 (12.2–41.3)
Specificity	100	100
PPV	100	100
NPV	69.7 (61.8–76.9)	81.6 (76.3–86.2)
A combination of PCR assays*		
Sensitivity	49.1 (35.4–62.9)	65.8 (47.4–82.4)
Specificity	100	97.9 (92.7–100)
PPV	100	88.9 (74.1–100)
NPV	79.1 (71.2–85.6)	91.0 (85.8–94.0)
**Nested 56kDa-based PCR assay**		
Sensitivity	40.0 (27.0–54.1)	56.8 (48.8–65.6)
Specificity	99.1 (94.9–100)	98.4 (96.7–100)
PPV	95.7 (78.1–99.9)	91.3 (78.3–100)
NPV	76.1 (68.1–82.9)	88.4 (83.3–92.0)
**47kDa-based real-time PCR assay**		
Sensitivity	47.3 (33.7–61.2)	63.2 (54.1–72.2)
Specificity	98.1 (93.4–99.8)	96.1 (93.2–98.4)
PPV	92.9 (76.5–99.1)	82.1 (67.9–92.9)
NPV	78.2 (70.2–84.9)	90.2 (85.0–93.2)
***GroEL*-based real-time PCR assay**		
Sensitivity	56.4 (42.3–69.7)	71.4 (62.5–80.0)
Specificity	96.2 (90.6–99.0)	93.0 (90.0–95.8)
PPV	88.6 (73.3–96.8)	74.3 (62.9–85.7)
NPV	81.0 (73.0–87.4)	92.1 (87.3–94.4)
IFA IgM**		
Sensitivity	83.6 (71.2–92.2)	70.0 (55.8–83.8)
Specificity	100	83.8 (76.4–90.0)
PPV	100	56.5 (47.8–65.2)
NPV	92.2 (85.7–96.4)	90.4 (85.2–94.8)
**Panbio ICT IgM**		
Sensitivity	47.3 (33.7–61.2)	72.8 (57.8–86.6)
Specificity	95.3 (89.3–98.5)	96.8 (91.7–99.7)
PPV	83.9 (66.3–94.5)	87.1 (77.4–100)
NPV	77.7 (69.6–84.5)	92.3 (86.9–96.2)
**Presence of eschar**		
Sensitivity	25.5 (14.7–39.0)	42.7 (26.4–61.1)
Specificity	97.2 (92.0–99.4)	98.9 (95.5–100)
PPV	82.4 (56.6–96.2)	94.1 (82.4–100)
NPV	71.5 (63.4–78.7)	85.4 (79.9–89.6)
A combination of PanBio ICT IgM and presence of eschar***		
Sensitivity	47.3 (33.7–61.2)	75.6 (65.1–85.7)
Specificity	93.4 (86.9–97.3)	95.9 (93.0–99.2)
PPV	78.8 (61.1–91.0)	84.9 (72.7–97.0)
NPV	77.3 (69.1–84.3)	93.0 (88.3–96.3)
A combination of *GroEL* PCR assay and PanBio ICT IgM ***		
Sensitivity	65.5 (51.4–77.8)	88.6 (79.0–94.4)
Specificity	92.5 (85.7–96.7)	90.8 (87.0–94.8)
PPV	81.8 (67.3–91.8)	75.0 (61.4–86.4)
NPV	83.8 (75.8–89.9)	96.6 (92.3–98.3)
A combination of *GroEL* PCR assay and presence of eschar***		
Sensitivity	61.8 (47.7–74.6)	84.6 (75.0–91.7)
Specificity	93.4 (86.9–97.3)	92.0 (88.6–95.1)
PPV	82.9 (67.9–92.8)	75.6 (63.4–85.4)
NPV	82.5 (74.5–88.8)	95.0 (90.8–97.5)

STIC is considered positive if either (a) *O*. *tsutsugamushi* is isolated, (b) at least two out of three PCR assays targeting the 56kDa, 47kDa and *groEL* genes are positive, (c) an admission IFA IgM titre is ≥ 1:12,800 or (d) there is at least a four-fold rise in convalescence IFA IgM titre compared to the admission IFA IgM titre [17,18].

^†^Values are means with 95% confidence interval.

^††^Values are medians with 95% credible interval.

*A combination of PCR assays was defined as positive when at least two out of the three PCR assays (56kDa PCR assay, 47kDa-based real-time PCR assay and *groEL*-based real-time PCR assays) were positive.

** IFA IgM was defined as positive in those with either admission IFA IgM titre of ≥1: 12,800 or at least a four-fold rise in convalescence IFA IgM titre compared to the admission IFA IgM titre.

*** A combination of two diagnostic tests was considered positive if either one of those tests was positive.

### Bayesian LCMs

Bayesian LCMs were then applied to obtain an unbiased estimate of the accuracy of each diagnostic test. There was no substantial difference between all Bayesian LCMs used (S2 Table). The model which took account of correlation between serological tests (IFA IgM and ICT IgM) had the lowest AIC and was selected as the best-fitting model.

True accuracies of diagnostic tests for scrub typhus estimated by the Bayesian LCM were significantly different from those estimated when assuming that STIC is perfect (Table 1). The Bayesian LCM estimated that STIC was an imperfect diagnostic test with a sensitivity of 90.5% and a specificity of 82.5%. The Bayesian LCM indicated that the prevalence of scrub typhus was actually much lower than estimated by relying on STIC, as there were a number of false positive results due to the low specificity of the IFA IgM (83.8%). In contrast, the true sensitivity of culture (24.4%), nested 56kDa PCR assay (56.7%), 47kDa-based real-time PCR assay (63.2%), *GroEL*-based real-time PCR assay (71.4%), PanBio ICT IgM (72.8%), and presence of eschar (42.7%) estimated by the Bayesian LCM were much higher than those estimated by assuming that STIC were perfect (Table 1). The specificity of Panbio ICT IgM was significantly higher than that of IFA (96.8% vs. 83.8%, p<0.001), but there was no significant difference in their sensitivities (72.8% vs. 70.0%, p = 0.34).

Because of the high diagnostic accuracy of the ICT IgM and the presence of an eschar, and the potential use of this combination at the bedside, we used the model to estimate the accuracy of a combination of both diagnostic tests. Using positive results from either test as indicating a positive diagnosis, the sensitivity and specificity of this combination were respectively 75.6% (95%CrI: 65.1–85.7%) and 95.9% (95%CrI: 93.0–99.2%, Table 1). Due to the high sensitivity of the *GroEL* PCR assay, we also estimated the accuracy of a combination of this PCR assay with either ICT IgM or with the presence of an eschar. Using positive results from either *GroEL* PCR assay or ICT IgM as indicating a positive diagnosis, the sensitivity and specificity of this combination were 88.6% (95%CrI: 79.0–94.4%) and 90.8% (95%CrI: 93.0–94.8%, Table 1), respectively.

### Post-hoc model evaluation

We could not identify any other data sets that were available and applicable for re-analysis using Bayesian LCMs. The naïve sensitivity of each diagnostic test estimated in only those who were culture, PCR assays or eschar positive is shown in Table 2. The naïve sensitivities of the IFA IgM in all groups were comparable to that estimated by the Bayesian LCM, and none were as high as the 83.6% estimated under the assumption that STIC is a perfect reference. This indicated that the low sensitivity of IFA IgM estimated by the Bayesian LCM was credible. We also evaluated the positivity of IFA IgM for scrub typhus in those who had alternative diagnoses. Of 10 murine typhus patients, 4 (40%) were IFA IgM positive, while culture, PCR assays and IgM ICT were all negative. Of 24 dengue patients, 5 (21%) were IFA IgM positive, while culture, PCR assays, and IgM ICT were all negative. This indicated that the low specificity of the IFA IgM in our setting was also credible.

**Table 2:** Naïve sensitivity of diagnostic tests estimated in those who had blood culture positive for *O*. *tsutsugamushi*, had a combination of PCR assays positive or had an eschar.

Population (n total)	56kDa-based PCR assays	47kDa-based PCR assay	*GroEL*-based PCR assay	IFA IgM *	Panbio ICT IgM	Presence of an eschar
Patients who had blood culture positive (n = 9)	66.7% (n = 6)	66.7% (n = 6)	77.8% (n = 7)	66.7% (n = 6)	55.6% (n = 5)	44.4% (n = 4)
Patients who had a combination of PCR assays positive (n = 27)**	81.5% (n = 22)	96.3% (n = 26)	100% (n = 27)	77.8% (n = 21)	74.1% (n = 20)	40.7% (n = 11)
Patients who had an eschar (n = 17)	64.7% (n = 11)	58.8% (n = 10)	64.7% (n = 11)	76.5% (n = 13)	88.3% (n = 15)	Not applicable

Data are sensitivity (n positive).

* IFA IgM was defined as positive in those with either admission IFA IgM titre of ≥1: 12,800 or at least a four-fold rise in convalescence IFA IgM titre compared to the admission IFA IgM titre.

** A combination of PCR assays was defined as positive when at least two out of the three PCR assays (56kDa PCR assay, 47kDa-based real-time PCR assay and *groEL*-based real-time PCR assays) were positive.

### Sensitivity analysis

Although sensitivities of diagnostic tests estimated by the Bayesian LCMs were minimally different when different priors were used, there was no substantial difference in specificities of diagnostic tests estimated and in the main outcomes of the analysis (S3 Table). All models estimated that IFA IgM had low sensitivity and low specificity, and that specificity of ICT IgM was significantly higher than that of IFA.

## Discussion

The key finding of this study is that the sensitivity and specificity of current reference tests for scrub typhus (IFA IgM and STIC (which includes the IFA IgM as one of the criteria)) are low. We confirmed this finding by performing a sophisticated statistical analysis, which did not a priori assume that either IFA IgM or STIC was a gold standard test with 100% sensitivity and 100% specificity. In addition, we also observed that a prototype IgM ICT had a better performance than IFA IgM. We demonstrated that the gold standard model inaccurately estimated the accuracy of the alternative diagnostic tests, and suggest that further evaluation of new diagnostic tests for scrub typhus should be performed with appropriate statistical models. Our study also has an implication for clinical practice. Our new finding supports the usage of a combination of IgM ICT assay and presence of eschar as a point-of-care diagnostic test for scrub typhus in resource-limited settings where PCR for scrub typhus is not part of standard of care. Various combinations of PCR, IgM ICT and presence of eschar could also be considered as a point-of-care diagnostic test for scrub typhus in developed countries.

To the best of our knowledge, this is the first study to systematically evaluate the accuracy of each diagnostic test and of reference tests for scrub typhus using Bayesian LCMs. We validated the low sensitivity of IFA by determining naïve sensitivities of each diagnostic test within different strata of the disease; the method that is also recommended to be used in the absence of a gold standard [23]. The low sensitivity of IFA IgM observed in all strata of the disease evaluated supports that the true sensitivity of IFA IgM is low. We also validated the low specificity of IFA IgM by checking the potential for false positivity of IFA IgM in patients who had a diagnosis of murine typhus or dengue infection in our study. We found that IFA IgM was commonly positive alone in those patients, and this supports the estimates obtained by our Bayesian LCM.

The finding that IFA IgM has a low sensitivity is neither novel nor surprising since IFA IgM have been found to be negative in scrub typhus patients who are blood culture positive for *O*. *tsutsugamushi* or PCR assay positive [8]. This is presumably because antibodies to *O*. *tsutsugamushi* may take several weeks to become detectable by IFA, and some patients might also fail to develop the antibodies against the organism even after a long period following infection [8]. Furthermore, significant variation in the antigenic composition of the IFA slides and the *O*. *tsutsugamushi* strain causing the infection may also contribute to this lack of accuracy.

The confirmation that IFA IgM has a low specificity is alarming. A high rate of false positivity provided by IFA IgM has long been suspected [8,16,32,33]. The major weaknesses of IFA include the reading of the fluorescent-stained slides, and the arbitrary cut-off limits for scrub typhus diagnosis [8,16,34]. When reading IFA slides determination of the fluorescence endpoint is based on the judgement of microscopists; therefore, the test is inherently subjective [16,35]. Dual infection with scrub typhus and other infectious diseases are found by serological tests to be common [33], but reports of dual infection by culture or molecular detection of both pathogens are rare [36]. These suggest that a proportion of the dual infections reported might actually be caused by false positivity or cross-reactivity of the IFA IgM. The plausible explanation is that infections caused by other organisms in our setting, such as murine typhus, may lead to production of IgM that reacts with the *O*. *tsutsugamushi* used in the IFA IgM assay [36]. The low specificity of the IFA IgM leads to the poor specificity of STIC. This is because STIC is considered positive if either culture is positive, at least two out of three PCR assays are positive, or IFA IgM is positive. Thus any false positivity due to IFA IgM will also lead to a false positivity of STIC.

A major effect of the low accuracy of STIC is that the accuracies of alternative diagnostic tests have been inaccurately estimated. The true sensitivities of blood culture, individual PCR assays, Panbio ICT IgM and presence of an eschar are much higher than those estimated by assuming that STIC is a perfect reference. The Panbio ICT IgM was erroneously discarded and not further developed into the market because of its apparent low sensitivity (Personal communication, SDB). Sensitivity of the Panbio ICT IgM was reported as 47.3% by comparing with STIC as a gold standard [17,18]. In this study, we show that the prototype ICT IgM developed by the Panbio Company actually has a high sensitivity and a higher specificity than the IFA IgM in our setting. Our study also suggests that ICT IgM might now be considered for use as a point-of-care test. For example, a combination of the Panbio ICT IgM and presence of an eschar has a PPV and NPV of 84.9% and 93.0% respectively, representing a test that could be used to diagnose scrub typhus with a moderately high degree of accuracy in our setting.

The high diagnostic accuracy of the ICT IgM could be due to the different antigen used in the test [13]. The ICT IgM used a truncated recombinant 56-kDa protein from a single *O*. *tsutsugamushi* Karp strain, which was purified and may explain the high specificity of PanBio ICT [28]. The IFA IgM used whole-cell antigen from three strains of *O*. *tsutsugamushi* (the Karp, Kato and Gilliam strains), and these whole-cell antigens may cross react to antibodies from other diseases. We found that although ICT IgM used the 56-kDa protein from a single Karp strain, the sensitivity of ICT IgM is comparable to the IFA IgM which used whole-cell antigen from the Karp, Kato and Gilliam strains. It is possible that the ICT IgM is also positive for scrub typhus patients infected with strains other than Karp. This is supported by the previous study showing that the 56-kDa protein from a Karp strain reacts to immune serum of mice infected with Kato, Gilliam and other strains [37].

The high diagnostic accuracy of presence of an eschar in our study could be explained because other diseases that can cause eschars, such as anthrax and spotted fever group rickettsioses are not common in Thailand [38]. Brown spider bites may cause wounds with identical appearance as an eschar; however, the scrub typhus-related eschar is painless. Therefore, it is still recommended that clinicians in scrub typhus endemic areas conduct an intensive search for painless eschars in patients with febrile illness.

This study has some limitations. The sample size in our study was small. We could not identify other data sets of suspected scrub typhus to prove whether the result observed in our study is generalisable in different settings. We could not evaluate the effects of other factors such as duration of symptoms, antimicrobial received prior to presentation, and different cut-off values of IFA IgM in our model.

We conclude that the current reference tests (STIC and IFA IgM) are imperfect in our setting, and there is an urgent need for improvement of serological reference tests for scrub typhus. Future evaluation of alternative diagnostic tests for acute scrub typhus should be done using a carefully selected set of diagnostic tests and appropriate statistical models.

## Supporting Information

S1 Table Description and model selection criteria. (DOCX)

S2 Table Prevalence, sensitivities and specificities for diagnostic tests for scrub typhus estimated by Bayesian latent class models. (DOCX)

S3 Table Prevalence, sensitivities and specificities for a diagnosis of scrub typhus using Bayesian latent class models with non-informative, sceptical and enthusiastic priors. (DOCX)

S1 Text The data set for Bayesian latent class models. (DOCX)

S2 Text WinBUGS models. (DOCX)
