# Supplementary material for: How to Determine the Accuracy of an Alternative Diagnostic Test when It Is Actually Better than the Reference Tests: A Re-Evaluation of Diagnostic Tests for Scrub Typhus Using Bayesian LCMs
Source: PLoS One. 2015 May 29;10(5):e0114930. doi: 10.1371/journal.pone.0114930 (PMC4449177; doi:10.1371/journal.pone.0114930)
Supplement: S1 Table — (DOCX) [file pone.0114930.s001.docx]

**Table S1. Description and model selection criteria**

| **Model No.** | **Correlation ^a^** | **Number of parameters ^b^** | **AIC ^c^** |
| --- | --- | --- | --- |
| 0 | None | 10 | 100.8 |
| 1 | Blood culture for *O. tsutsugamushi* and a combination of PCR assays | 11 | 103.4 |
| 2 | IFA IgM and PanBio ICT IgM | 11 | 99.2 |

**^a^** All correlations are in infected subjects.

**^b^** Number of parameters is the total number of unknown parameters to be estimated. For example, the number 10 for Model 0 represented prevalence (1), sensitivity of blood culture (1) and sensitivities and specificities (8) of four serological tests (Specificity of culture is fixed at 100%).

**^c^** AIC (Akaike information criteria) is the number representing the goodness of fit of an estimated statistical model. The model with the smallest AIC was estimated to be the model that would best predict a replicate dataset, which had the same structure as that actually observed. A difference in AIC of more than 10 indicated definite support to the model with the lower AIC, while a difference of between 5 and 10 was considered substantial, and less than 5 inconclusive
