# Supplementary material for: How to Determine the Accuracy of an Alternative Diagnostic Test when It Is Actually Better than the Reference Tests: A Re-Evaluation of Diagnostic Tests for Scrub Typhus Using Bayesian LCMs
Source: PLoS One. 2015 May 29;10(5):e0114930. doi: 10.1371/journal.pone.0114930 (PMC4449177; doi:10.1371/journal.pone.0114930)
Supplement: S2 Table — (DOCX) [file pone.0114930.s002.docx]

**Table S2. Prevalence, sensitivities and specificities of diagnostic tests for scrub typhus estimated by Bayesian latent class models**

| **Parameters** | **STIC as gold standard**  **(95% CI) ^a^** | **Model 0**  **(95% CrI) ^b^** | **Model 1**  **(95% CrI) ^b^** | **Final Bayesian LCM**  **(95% CrI) ^b^** |
| --- | --- | --- | --- | --- |
| **Prevalence** | 34.2 (26.9-42.0) | 22.7 (15.7-31.2) | 23.2 (15.9-31.9) | 23.0 (15.9-31.5) |
| **STIC** |  |  |  |  |
| Sensitivity | 100 | 91.9 (82.9-100) | 91.4 (81.8-97.4) | 90.5 (79.6-100) |
| Specificity | 100 | 82.7 (80.0-86.2) | 82.8 (80.0-86.6) | 82.5 (79.4-85.6) |
| **Blood culture for *O. tsustsugamushi*** |  |  |  |  |
| Sensitivity | 16.4 (7.8-28.8) | 24.6 (12.3-41.1) | 24.1 (12.5-40.1) | 24.4 (12.2-41.3) |
| Specificity | 100 | 100 | 100 | 100 |
| **A combination of PCR assays ^c^** |  |  |  |  |
| Sensitivity | 49.1 (35.4-62.9) | 65.6 (47.7-82.5) | 64.5 (47.1-80.6) | 65.8 (47.4-82.4) |
| Specificity | 100 | 97.7 (92.9-99.9) | 97.6 (92.9-99.9) | 97.9 (92.7-100.0) |
| **Nested 56kDa-based PCR assay** |  |  |  |  |
| Sensitivity | 40.0 (27.0-54.1) | 57.9 (50.0-66.7) | 56.8 (48.8-65.6) | 56.8 (48.8-65.6) |
| Specificity | 99.1 (94.9-100.0) | 98.4 (96.9-100) | 98.4 (96.9-100) | 98.4 (96.7-100.0) |
| **47kDa-based real-time PCR assay** |  |  |  |  |
| Sensitivity | 47.3 (33.7-61.2) | 62.9 (53.9-71.9) | 61.9 (53.1-71.0) | 63.2 (53.7-72.2) |
| Specificity | 98.1 (93.4-99.8) | 96.0 (93.7-98.4) | 96.0 (93.7-98.4) | 96.1 (93.2-98.4) |
| ***GroEL-*based real-time PCR assay** |  |  |  |  |
| Sensitivity | 56.4 (42.3-69.7) | 72.2 (63.4-71.9) | 71.1 (61.9-79.4) | 71.4 (62.5-80.0) |
| Specificity | 96.2 (90.6-99.0) | 92.9 (90.6-95.7) | 93.0 (90.6-95.8) | 93.0 (90.0-95.8) |
| **IFA IgM ^d^** |  |  |  |  |
| Sensitivity | 83.6 (71.2-92.2) | 73.8 (56.3-88.1) | 73.0 (55.5-87.8) | 70.0 (55.8-83.8) |
| Specificity | 100 | 84.6 (77.3-90.7) | 84.8 (77.3-91.1) | 84.0 (76.3-90.2) |
| **PanBio ICT IgM** |  |  |  |  |
| Sensitivity | 47.3 (33.7-61.2) | 76.1 (57.3-90.6) | 75.2 (56.9-90.0) | 72.8 (57.8-86.6) |
| Specificity | 95.3 (89.3-98.5) | 97.3 (92.3-99.9) | 97.6 (92.5-99.9) | 96.8 (91.7-99.7) |
| **Presence of eschar** |  |  |  |  |
| Sensitivity | 25.5 (14.7-39.0) | 41.7 (25.8-60.2) | 41.4 (25.3-60.1) | 42.7 (26.4-61.1) |
| Specificity | 97.2 (92.0-99.4) | 98.5 (95.2-99.9) | 98.6 (95.2-99.9) | 98.9 (95.5-100.0) |
| **Combination of PanBio ICT IgM and presence of eschar ^e^** |  |  |  |  |
| Sensitivity | 47.3 (33.7-61.2) | 77.1 (67.4-85.3) | 76.5 (66.0-85.3) | 75.6 (65.1-85.7) |
| Specificity | 93.4 (86.9-97.3) | 96.0 (93.1-99.2) | 96.1 (93.5-99.2) | 95.9 (93.0-99.2) |
| **Combination of *GroEL*-based real-time PCR assay and PanBio ICT IgM ^f^** |  |  |  |  |
| Sensitivity | 65.5 (51.4-77.8) | 90.9 (81.3-94.6) | 89.7 (79.6-94.6) | 88.6 (79.0-94.4) |
| Specificity | 92.5 (85.7-96.7) | 91.1 (87.7-94.9) | 91.3 (87.8-95.0) | 90.8 (87.0-94.8) |
| **Combination of *GroEL*-based assay and presence of eschar ^f^** |  |  |  |  |
| Sensitivity | 61.8 (47.7-74.6) | 83.8 (74.4-91.4) | 82.9 (72.9-90.9) | 84.6 (75.0-91.7) |
| Specificity | 93.4 (86.9-97.3) | 91.7 (88.9-94.8) | 91.9 (89.1-94.9) | 92.0 (88.6-95.1) |

STIC is considered positive if either (a) *O. tsutsugamushi* is isolated, (b) at least two out of three PCR assays targeting the 56kDa, 47kDa and *groEL* genes are positive, (c) an admission IFA IgM titre is ≥ 1:12,800 or (d) there is at least a four-fold rise in convalescence IFA IgM titre compared to the admission IFA IgM titre [[17](#_ENREF_17),[18](#_ENREF_18)].

^a^ Values are means of estimates with 95% confidence interval.

^b^ Values are medians of estimates with 95% credible interval.

^c^ A combination of PCR assays was defined as positive when at least two out of the three PCR assays (nested 56kDa PCR assay, 47kDa-based real-time PCR assay and *groEL*-based real-time PCR assays) were positive.

^d^ IFA IgM was defined as positive in those with either admission IFA IgM titre of ≥1: 12,800 or at least a four-fold rise in convalescence IFA IgM titre compared to the admission IFA IgM titre.

^e^ A combination of PanBio ICT IgM and presence of eschar was defined as positive in those with either ICT IgM had positive result or eschar was identified in the clinical setting.

^f^ A combination of two diagnostic tests was considered positive if either one of those tests was positive.
