# Supplementary material for: How to Determine the Accuracy of an Alternative Diagnostic Test when It Is Actually Better than the Reference Tests: A Re-Evaluation of Diagnostic Tests for Scrub Typhus Using Bayesian LCMs
Source: PLoS One. 2015 May 29;10(5):e0114930. doi: 10.1371/journal.pone.0114930 (PMC4449177; doi:10.1371/journal.pone.0114930)
Supplement: S3 Table — (DOCX) [file pone.0114930.s003.docx]

**Table S3. Prevalence, sensitivities and specificities for a diagnosis of scrub typhus using Bayesian latent class models with non-informative, sceptical and enthusiastic priors.**

| **Parameters** | **Bayesian model with non-informative priors**  **(95% CrI) ^a^** | **Bayesian model with sceptical**  **priors**  **(95% CrI) ^b^** | **Bayesian model with enthusiastic**  **priors**  **(95% CrI) ^c^** |
| --- | --- | --- | --- |
| **Prevalence** | 23.0 (15.9-31.5) | 20.6 (13.9-28.5) | 21.1 (14.3-29.3) |
| **STIC** |  |  |  |
| Sensitivity | 90.5 (79.6-100) | 93.6 (82.1-100) | 93.8 (84.2-100) |
| Specificity | 82.5 (79.4-85.6) | 80.9 (78.2-84.0) | 81.5 (78.8-84.6) |
| **Blood culture for *O. tsutsugamushi*** |  |  |  |
| Sensitivity | 24.4 (12.2-41.3) | 29.5 (16.2-46.7) | 30.7 (16.4-48.2) |
| Specificity | 100 | 100 | 100 |
| **A combination of PCR assays ^d^** |  |  |  |
| Sensitivity | 65.8 (47.4-82.4) | 66.0 (48.5-81.7) | 71.0 (53.2-86.1) |
| Specificity | 97.9 (92.7-100.0) | 94.4 (89.2-97.8) | 97.0 (92.2-99.7) |
| **Nested 56kDa-based PCR assay** |  |  |  |
| Sensitivity | 56.8 (48.8-65.6) | 60.0 (51.2-69.2) | 59.5 (52.4-67.9) |
| Specificity | 98.4 (96.7-100.0) | 97.6 (96.0-99.2) | 97.7 (96.2-99.2) |
| **47kDa-based real-time PCR assay** |  |  |  |
| Sensitivity | 63.2 (53.7-72.2) | 64.7 (55.3-73.3) | 65.5 (57.1-73.3) |
| Specificity | 96.1 (93.2-98.4) | 94.7 (92.4-97.6) | 95.3 (93.0-98.4) |
| ***GroEL-*based real-time PCR assay** |  |  |  |
| Sensitivity | 71.4 (62.5-80.0) | 73.3 (63.4-81.8) | 73.7 (65.7-81.3) |
| Specificity | 93.0 (90.0-95.8) | 91.5 (88.9-94.3) | 92.1 (89.5-95.1) |
| **IFA IgM ^e^** |  |  |  |
| Sensitivity | 70.0 (55.8-83.8) | 71.0 (57.5-83.7) | 74.3 (61.6-86.7) |
| Specificity | 84.0 (76.3-90.2) | 82.1 (74.8-88.3) | 84.0 (76.9-90.0) |
| **PanBio ICT IgM** |  |  |  |
| Sensitivity | 72.8 (57.8-86.6) | 72.2 (58.7-84.8) | 77.2 (63.5-89.8) |
| Specificity | 96.8 (91.7-99.7) | 93.4 (88.0-97.1) | 96.3 (91.4-99.1) |
| **Presence of eschar** |  |  |  |
| Sensitivity | 42.7 (26.4-61.1) | 45.4 (29.0-63.7) | 48.8 (31.5-67.4) |
| Specificity | 98.9 (95.5-100.0) | 96.1 (91.9-98.5) | 98.5 (95.4-99.8) |
| **Combination of PanBio ICT IgM and presence of eschar ^f^** |  |  |  |
| Sensitivity | 75.6 (65.1-85.7) | 76.9 (66.7-85.7) | 78.8 (69.1-87.1) |
| Specificity | 95.9 (93.0-99.2) | 93.9 (91.4-96.8) | 95.2 (92.4-97.6) |
| **Combination of *GroEL*-based real-time PCR assay and PanBio ICT IgM ^f^** |  |  |  |
| Sensitivity | 88.6 (79.0-94.4) | 88.6 (78.4-93.9) | 90.6 (82.5-94.3) |
| Specificity | 90.8 (87.0-94.8) | 88.4 (85.2-91.8) | 89.5 (86.3-92.7) |
| **Combination of *GroEL*-based real-time PCR assay and presence of eschar ^f^** |  |  |  |
| Sensitivity | 84.6 (75.0-91.7) | 85.3 (75.0-93.1) | 86.8 (79.0-93.1) |
| Specificity | 92.0 (88.6-95.1) | 89.8 (87.1-92.8) | 90.6 (87.9-94.2) |

STIC is considered positive if either (a) *O. tsutsugamushi* is isolated, (b) at least two out of three PCR assays targeting the 56kDa, 47kDa and *groEL* genes are positive, (c) an admission IFA IgM titre is ≥ 1:12,800 or (d) there is at least a four-fold rise in convalescence IFA IgM titre compared to the admission IFA IgM titre [[17](#_ENREF_17),[18](#_ENREF_18)].

^a^ Inverse logit transformation of normal distribution (0.0, 0.1) was selected as non-informative priors and used for sensitivities and specificities of every test. This prior assumed that no prior information (non-informative priors) about the unknown parameters (prevalence, sensitivities and specificities) was available, except that the specificity of culture was fixed at 100%.

^b^ Inverse logit transformation of normal distribution (0.0, 1.0) was selected as sceptical priors and used for sensitivities and specificities of every test, except that the specificity of culture was fixed at 100%. This prior strongly believed that sensitivity and specificity of each test is around 50%, and there is less than 1% chance that sensitivity and specificity of each test is more than 95% or less than 5%.

^c^ Inverse logit transformation of normal distribution (3.0, 0.4) was selected as enthusiastic priors and used for sensitivities and specificities of every test, except that the specificity of culture was fixed at 100%. This prior strongly believed that sensitivity and specificity of each test is close to 95%, and there is less than 3% chance that sensitivity and specificity of each test is less than 50%.

^d^ A combination of PCR assays was defined as positive when at least two out of the three PCR assays (nested 56kDa PCR assay, 47kDa-based real-time PCR assay and *groEL*-based real-time PCR assays) were positive.

^e^ IFA IgM was defined as positive in those with either admission IFA IgM titre of ≥1: 12,800 or at least a four-fold rise in convalescence IFA IgM titre compared to the admission IFA IgM titre.

^f^ A combination of PanBio ICT IgM and presence of eschar was defined as positive in those with either ICT IgM had positive result or eschar was identified in the clinical setting.
