# Supplementary material for: How to Determine the Accuracy of an Alternative Diagnostic Test when It Is Actually Better than the Reference Tests: A Re-Evaluation of Diagnostic Tests for Scrub Typhus Using Bayesian LCMs
Source: PLoS One. 2015 May 29;10(5):e0114930. doi: 10.1371/journal.pone.0114930 (PMC4449177; doi:10.1371/journal.pone.0114930)
Supplement: S1 Text — (DOCX) [file pone.0114930.s004.docx]

**Supporting information**

**Text S1. The data set for Bayesian latent class models**

This is the data set for Bayesian latent class models. The first, second, third, fourth, and fifth columns are the results of blood culture for *O. tsutsugamushi*, a combination of PCR assays, IFA IgM, Panbio ICT IgM, and presence of an eschar. The sixth, seventh, eighth, ninth, tenth, eleventh and twelfth columns are the results of nested 56kDa-based PCR assay, 47kDa-based real-time PCR assay, *GroEL*-based real-time PCR assay, STIC, a combination of PanBio ICT IgM and presence of eschar, a combination of *GroEL*-based real-time PCR assay and PanBio ICT IgM, and a combination of *GroEL*-based real-time PCR assay and presence of eschar, respectively: 1 = positive result and 0 = negative result. Each row represents each participant.

# Data

list(y=structure(

.Data=c(

0, 0, 0, 0, 0, 0, 0, 0, 0, 0, 0, 0,

0, 1, 1, 1, 1, 1, 1, 1, 1, 1, 1, 1,

0, 0, 0, 0, 0, 0, 0, 0, 0, 0, 0, 0,

0, 0, 1, 1, 1, 0, 0, 0, 1, 1, 1, 1,

0, 0, 0, 1, 1, 0, 0, 0, 0, 1, 1, 1,

0, 0, 1, 1, 1, 0, 0, 0, 1, 1, 1, 1,

0, 0, 0, 0, 0, 0, 0, 1, 0, 0, 1, 1,

0, 0, 1, 1, 0, 0, 0, 1, 1, 1, 1, 1,

1, 1, 1, 1, 1, 1, 1, 1, 1, 1, 1, 1,

0, 0, 0, 0, 0, 0, 0, 0, 0, 0, 0, 0,

0, 0, 0, 0, 0, 0, 0, 0, 0, 0, 0, 0,

0, 0, 0, 1, 0, 0, 0, 0, 0, 1, 1, 0,

0, 0, 0, 1, 0, 0, 0, 1, 0, 1, 1, 1,

0, 1, 1, 1, 0, 1, 1, 1, 1, 1, 1, 1,

1, 1, 1, 1, 1, 1, 1, 1, 1, 1, 1, 1,

0, 0, 0, 0, 0, 0, 0, 1, 0, 0, 1, 1,

0, 1, 0, 1, 0, 0, 1, 1, 1, 1, 1, 1,

0, 1, 0, 0, 0, 0, 1, 1, 1, 0, 1, 1,

0, 0, 0, 0, 0, 0, 0, 0, 0, 0, 0, 0,

0, 0, 0, 0, 0, 0, 0, 0, 0, 0, 0, 0,

0, 0, 1, 0, 0, 0, 0, 0, 1, 0, 0, 0,

0, 0, 0, 0, 0, 0, 0, 0, 0, 0, 0, 0,

0, 0, 0, 0, 0, 0, 0, 1, 0, 0, 1, 1,

1, 0, 0, 0, 0, 0, 0, 1, 1, 0, 1, 1,

0, 0, 0, 0, 0, 0, 0, 0, 0, 0, 0, 0,

0, 0, 0, 0, 0, 0, 0, 0, 0, 0, 0, 0,

0, 0, 0, 0, 0, 0, 0, 0, 0, 0, 0, 0,

0, 0, 1, 0, 0, 0, 0, 1, 1, 0, 1, 1,

0, 0, 0, 0, 0, 0, 0, 0, 0, 0, 0, 0,

0, 0, 0, 0, 0, 0, 0, 0, 0, 0, 0, 0,

0, 0, 0, 0, 0, 0, 0, 0, 0, 0, 0, 0,

0, 1, 0, 1, 0, 1, 1, 1, 1, 1, 1, 1,

0, 0, 0, 0, 0, 0, 0, 0, 0, 0, 0, 0,

0, 0, 0, 0, 0, 0, 0, 0, 0, 0, 0, 0,

0, 1, 1, 0, 0, 0, 1, 1, 1, 0, 1, 1,

1, 1, 1, 1, 1, 1, 1, 1, 1, 1, 1, 1,

0, 0, 0, 0, 0, 0, 0, 0, 0, 0, 0, 0,

0, 1, 1, 0, 0, 1, 1, 1, 1, 0, 1, 1,

0, 0, 0, 0, 0, 0, 0, 0, 0, 0, 0, 0,

0, 0, 0, 0, 0, 0, 0, 0, 0, 0, 0, 0,

0, 0, 0, 0, 0, 0, 0, 0, 0, 0, 0, 0,

0, 0, 1, 1, 1, 0, 0, 0, 1, 1, 1, 1,

0, 0, 0, 0, 0, 0, 0, 0, 0, 0, 0, 0,

0, 0, 0, 0, 0, 0, 0, 0, 0, 0, 0, 0,

0, 0, 1, 0, 0, 0, 0, 0, 1, 0, 0, 0,

0, 0, 0, 0, 0, 0, 0, 0, 0, 0, 0, 0,

0, 1, 0, 1, 1, 1, 1, 1, 1, 1, 1, 1,

0, 0, 0, 0, 0, 0, 0, 0, 0, 0, 0, 0,

0, 0, 0, 0, 0, 0, 0, 0, 0, 0, 0, 0,

0, 0, 0, 0, 0, 0, 0, 0, 0, 0, 0, 0,

0, 0, 0, 0, 0, 0, 0, 0, 0, 0, 0, 0,

0, 0, 0, 0, 1, 0, 0, 0, 0, 1, 0, 1,

1, 0, 0, 0, 0, 0, 0, 0, 1, 0, 0, 0,

0, 0, 1, 1, 0, 0, 0, 0, 1, 1, 1, 0,

0, 0, 0, 0, 0, 0, 0, 0, 0, 0, 0, 0,

0, 0, 0, 0, 0, 0, 0, 0, 0, 0, 0, 0,

1, 1, 1, 1, 1, 1, 1, 1, 1, 1, 1, 1,

0, 0, 0, 0, 0, 0, 0, 0, 0, 0, 0, 0,

0, 0, 0, 0, 0, 0, 0, 0, 0, 0, 0, 0,

0, 0, 0, 0, 0, 0, 0, 0, 0, 0, 0, 0,

0, 0, 0, 0, 0, 0, 0, 0, 0, 0, 0, 0,

0, 0, 0, 0, 0, 0, 0, 0, 0, 0, 0, 0,

0, 0, 0, 0, 0, 0, 0, 0, 0, 0, 0, 0,

0, 0, 0, 0, 0, 0, 0, 0, 0, 0, 0, 0,

0, 0, 0, 0, 0, 0, 0, 0, 0, 0, 0, 0,

0, 0, 0, 0, 0, 0, 0, 0, 0, 0, 0, 0,

0, 0, 0, 0, 0, 0, 0, 0, 0, 0, 0, 0,

0, 0, 0, 0, 0, 0, 0, 0, 0, 0, 0, 0,

0, 0, 0, 0, 0, 0, 0, 0, 0, 0, 0, 0,

0, 0, 0, 0, 0, 0, 0, 0, 0, 0, 0, 0,

0, 1, 1, 1, 0, 1, 1, 1, 1, 1, 1, 1,

0, 0, 0, 0, 0, 0, 0, 0, 0, 0, 0, 0,

0, 0, 0, 0, 0, 0, 0, 0, 0, 0, 0, 0,

0, 0, 0, 0, 0, 0, 0, 0, 0, 0, 0, 0,

0, 1, 1, 1, 0, 0, 1, 1, 1, 1, 1, 1,

0, 0, 0, 0, 0, 0, 0, 0, 0, 0, 0, 0,

0, 0, 0, 0, 0, 0, 0, 0, 0, 0, 0, 0,

0, 0, 0, 0, 0, 0, 0, 0, 0, 0, 0, 0,

0, 0, 1, 0, 0, 0, 0, 1, 1, 0, 1, 1,

0, 0, 0, 0, 0, 0, 0, 0, 0, 0, 0, 0,

0, 1, 1, 1, 0, 1, 1, 1, 1, 1, 1, 1,

0, 0, 0, 0, 0, 0, 0, 0, 0, 0, 0, 0,

0, 0, 0, 0, 0, 0, 0, 0, 0, 0, 0, 0,

0, 1, 0, 0, 0, 1, 1, 1, 1, 0, 1, 1,

0, 0, 0, 0, 0, 0, 0, 0, 0, 0, 0, 0,

0, 0, 0, 0, 0, 0, 0, 0, 0, 0, 0, 0,

0, 0, 1, 0, 0, 0, 0, 0, 1, 0, 0, 0,

0, 0, 0, 0, 0, 0, 0, 0, 0, 0, 0, 0,

0, 0, 0, 0, 0, 0, 0, 0, 0, 0, 0, 0,

0, 0, 0, 0, 0, 0, 0, 0, 0, 0, 0, 0,

0, 0, 1, 0, 0, 0, 0, 0, 1, 0, 0, 0,

0, 0, 0, 0, 0, 0, 0, 0, 0, 0, 0, 0,

0, 0, 0, 0, 0, 0, 0, 0, 0, 0, 0, 0,

0, 1, 1, 1, 1, 1, 1, 1, 1, 1, 1, 1,

0, 0, 1, 1, 0, 0, 0, 0, 1, 1, 1, 0,

0, 0, 1, 0, 0, 0, 0, 0, 1, 0, 0, 0,

0, 0, 0, 0, 0, 0, 0, 0, 0, 0, 0, 0,

0, 0, 0, 0, 0, 0, 0, 0, 0, 0, 0, 0,

0, 0, 0, 0, 0, 0, 0, 0, 0, 0, 0, 0,

0, 1, 1, 1, 0, 1, 1, 1, 1, 1, 1, 1,

0, 0, 1, 0, 0, 0, 0, 0, 1, 0, 0, 0,

0, 0, 1, 0, 0, 0, 0, 0, 1, 0, 0, 0,

0, 0, 1, 0, 0, 0, 0, 0, 1, 0, 0, 0,

0, 0, 0, 0, 0, 0, 0, 0, 0, 0, 0, 0,

0, 1, 1, 0, 0, 1, 1, 1, 1, 0, 1, 1,

0, 0, 0, 0, 0, 0, 0, 0, 0, 0, 0, 0,

0, 0, 1, 0, 0, 0, 0, 0, 1, 0, 0, 0,

0, 0, 1, 0, 0, 0, 0, 0, 1, 0, 0, 0,

0, 0, 0, 0, 0, 0, 0, 0, 0, 0, 0, 0,

0, 0, 1, 0, 0, 0, 0, 0, 1, 0, 0, 0,

0, 0, 0, 0, 0, 0, 0, 0, 0, 0, 0, 0,

0, 1, 0, 0, 0, 0, 1, 1, 1, 0, 1, 1,

0, 0, 0, 0, 0, 0, 0, 0, 0, 0, 0, 0,

1, 1, 1, 0, 0, 1, 1, 1, 1, 0, 1, 1,

0, 0, 0, 0, 0, 0, 0, 0, 0, 0, 0, 0,

0, 0, 0, 0, 0, 0, 0, 0, 0, 0, 0, 0,

0, 0, 0, 0, 0, 0, 0, 0, 0, 0, 0, 0,

0, 0, 0, 0, 0, 0, 0, 0, 0, 0, 0, 0,

0, 0, 0, 0, 0, 0, 0, 0, 0, 0, 0, 0,

0, 0, 0, 0, 0, 0, 0, 0, 0, 0, 0, 0,

0, 0, 0, 0, 0, 0, 0, 0, 0, 0, 0, 0,

0, 0, 0, 0, 0, 0, 0, 0, 0, 0, 0, 0,

0, 0, 1, 0, 0, 0, 0, 0, 1, 0, 0, 0,

0, 0, 1, 0, 0, 0, 0, 0, 1, 0, 0, 0,

0, 0, 1, 0, 0, 0, 0, 0, 1, 0, 0, 0,

0, 1, 1, 1, 1, 1, 1, 1, 1, 1, 1, 1,

0, 0, 0, 0, 0, 0, 1, 0, 0, 0, 0, 0,

0, 0, 0, 0, 0, 0, 0, 0, 0, 0, 0, 0,

0, 0, 0, 0, 0, 0, 0, 0, 0, 0, 0, 0,

0, 0, 0, 0, 0, 0, 0, 0, 0, 0, 0, 0,

0, 0, 0, 0, 0, 0, 1, 0, 0, 0, 0, 0,

0, 0, 0, 0, 0, 0, 0, 0, 0, 0, 0, 0,

0, 0, 0, 0, 0, 0, 0, 0, 0, 0, 0, 0,

0, 0, 0, 0, 0, 0, 0, 0, 0, 0, 0, 0,

0, 0, 0, 0, 0, 0, 0, 0, 0, 0, 0, 0,

1, 1, 1, 1, 0, 1, 1, 1, 1, 1, 1, 1,

1, 0, 0, 0, 0, 0, 0, 0, 1, 0, 0, 0,

0, 0, 0, 0, 0, 0, 0, 0, 0, 0, 0, 0,

0, 0, 0, 0, 0, 0, 0, 0, 0, 0, 0, 0,

0, 1, 1, 1, 1, 1, 0, 1, 1, 1, 1, 1,

0, 0, 0, 0, 0, 0, 0, 0, 0, 0, 0, 0,

0, 0, 0, 0, 0, 0, 0, 0, 0, 0, 0, 0,

0, 0, 0, 0, 0, 0, 0, 0, 0, 0, 0, 0,

0, 0, 0, 0, 0, 0, 0, 0, 0, 0, 0, 0,

0, 0, 1, 0, 0, 0, 0, 0, 1, 0, 0, 0,

0, 0, 1, 0, 0, 0, 0, 0, 1, 0, 0, 0,

0, 0, 0, 0, 0, 0, 0, 0, 0, 0, 0, 0,

0, 1, 1, 1, 1, 1, 1, 1, 1, 1, 1, 1,

0, 1, 1, 1, 0, 1, 1, 1, 1, 1, 1, 1,

0, 1, 1, 1, 1, 1, 1, 1, 1, 1, 1, 1,

0, 0, 0, 0, 0, 0, 0, 0, 0, 0, 0, 0,

0, 0, 0, 0, 1, 0, 0, 0, 0, 1, 0, 1,

0, 0, 0, 0, 0, 0, 0, 0, 0, 0, 0, 0,

0, 0, 1, 0, 0, 0, 0, 0, 1, 0, 0, 0,

0, 0, 0, 0, 0, 0, 0, 0, 0, 0, 0, 0,

0, 0, 0, 0, 0, 0, 0, 0, 0, 0, 0, 0,

0, 0, 0, 1, 0, 0, 0, 0, 0, 1, 1, 0,

0, 0, 0, 0, 0, 0, 0, 0, 0, 0, 0, 0,

0, 0, 0, 0, 0, 0, 0, 0, 0, 0, 0, 0,

0, 0, 0, 1, 0, 1, 0, 0, 0, 1, 1, 0,

0, 0, 0, 0, 0, 0, 0, 0, 0, 0, 0, 0

),.Dim=c(161,12)),

pattern=structure(

.Data=c(

1, 1, 1, 1, 1,

1, 1, 1, 1, 0,

1, 1, 1, 0, 1,

1, 1, 0, 1, 1,

1, 0, 1, 1, 1,

0, 1, 1, 1, 1,

1, 1, 1, 0, 0,

1, 1, 0, 1, 0,

1, 0, 1, 1, 0,

0, 1, 1, 1, 0,

1, 1, 0, 0, 1,

1, 0, 1, 0, 1,

0, 1, 1, 0, 1,

1, 0, 0, 1, 1,

0, 1, 0, 1, 1,

0, 0, 1, 1, 1,

1, 1, 0, 0, 0,

1, 0, 1, 0, 0,

1, 0, 0, 1, 0,

1, 0, 0, 0, 1,

0, 1, 1, 0, 0,

0, 1, 0, 1, 0,

0, 1, 0, 0, 1,

0, 0, 1, 1, 0,

0, 0, 1, 0, 1,

0, 0, 0, 1, 1,

1, 0, 0, 0, 0,

0, 1, 0, 0, 0,

0, 0, 1, 0, 0,

0, 0, 0, 1, 0,

0, 0, 0, 0, 1,

0, 0, 0, 0, 0

),.Dim=c(32,5)))
