# Supplementary material for: How to Determine the Accuracy of an Alternative Diagnostic Test when It Is Actually Better than the Reference Tests: A Re-Evaluation of Diagnostic Tests for Scrub Typhus Using Bayesian LCMs
Source: PLoS One. 2015 May 29;10(5):e0114930. doi: 10.1371/journal.pone.0114930 (PMC4449177; doi:10.1371/journal.pone.0114930)
Supplement: S2 Text — (DOCX) [file pone.0114930.s005.docx]

**Text S2. WinBUGS models**

**2.1 WinBUGS code for model 0**

# Conditional independence model (result of each diagnostic test on a given patient was independent conditional on true disease status of the patient)

model{

for (i in 1:161){

status[i]~dbern(prev)

# Likelihood was estimated by five diagnostic tests; including blood culture for *O. tsutsugamushi*, a combination of PCR assays, IFA IgM, Panbio ICT IgM, and presence of an eschar

for (j in 1:5){

y[i,j]~dbern(p[i,j])

ypred[i,j]~dbern(p[i,j])

logit(p[i,j])<-status[i]*alpha[j]+(1-status[i])*beta[j]

}

}

# Prior

prev~dbeta(0.5,0.5)

alpha[1]~dnorm(0.0,0.1)

logit(s[1])<-alpha[1]

beta[1]<--1000

for (j in 2:5){

alpha[j]~dnorm(0.0,0.1)

beta[j]~dnorm(0.0,0.1)I(,1)

logit(s[j])<-alpha[j]

logit(x[j])<--beta[j]

}

se[1]<-s[1]

se[2]<-s[2]

se[3]<-s[3]

se[4]<-s[4]

se[5]<-s[5]

# Using MCMC to estimate the accuracies of nested 56kDa PCR assay, 47kDa-based real-time PCR assay, *GroEL*-based real-time PCR assay, STIC, combined ICT IgM with presence of eschar, combined *GroEL-*based real-time PCR assay with PanBio ICT IgM, and combined *GroEL-*based real-time PCR assay with presence of eschar

for (i in 1:161){

for (j in 6:12){

tdiseasetestpos[i,j]<-status[i]*y[i,j]

tnondiseasetestpos[i,j]<-(1-status[i])*y[i,j]

}

}

for (j in 6:12){

se[j]<-sum(tdiseasetestpos[,j])/sum(status[])

x[j]<-1-sum(tnondiseasetestpos[,j])/(161-sum(status[]))

}

# Prediction

for (i in 1:161){

for (k in 1:32){

for (j in 1:5){

arraymatched[i,k,j]<-equals(ypred[i,j],pattern[k,j])

}

nmatched[i,k]<-sum(arraymatched[i,k,])

matchedpattern[i,k]<-equals(nmatched[i,k],5)

}

}

for (k in 1:32){

freqpred[k]<-sum(matchedpattern[,k])

}

#Bayesian p value

for (i in 1:161){

for (k in 1:32){

for (j in 1:5){

arraymatchedobs[i,k,j]<-equals(y[i,j],pattern[k,j])

}

nmatchedobs[i,k]<-sum(arraymatchedobs[i,k,])

matchedpatternobs[i,k]<-equals(nmatchedobs[i,k],5)

}

}

for (k in 1:32){

freqobs[k]<-sum(matchedpatternobs[,k])

}

for (k in 1:32){

pvalue[k]<-step(freqpred[k]-freqobs[k])

}

pvalueSe34<-step(se[4]-se[3])

pvalueSp34<-step(x[4]-x[3])

# PPV and NPV

for (i in 1:161){

for (j in 1:12){

testpos[i,j]<-y[i,j]

testneg[i,j]<-1-y[i,j]

diseasetestpos[i,j]<-status[i]*y[i,j]

nondiseasetestneg[i,j]<-(1-status[i])*(1-y[i,j])

}

}

for (j in 1:12){

stestpos[j]<-sum(testpos[,j])

stestneg[j]<-sum(testneg[,j])

sdiseasetestpos[j]<-sum(diseasetestpos[,j])

snondiseasetestneg[j]<-sum(nondiseasetestneg[,j])

ppv[j]<- sdiseasetestpos[j]/stestpos[j]

npv[j]<- snondiseasetestneg[j]/stestneg[j]

}

}

**2.2 WinBUGS code for Model 1**

# Conditional dependence model between blood culture for *O. tsutsugamushi* and a combination of PCR assays

model{

for (i in 1:161){

status[i]~dbern(prev)

for (j in 1:5){

y[i,j]~dbern(p[i,j])

ypred[i,j]~dbern(p[i,j])

}

}

for (i in 1:161){

for (j in 3:4){

logit(p[i,j])<-status[i]*alpha[j]+(1-status[i])*beta[j]

}

for (j in 1:2){

logit(p[i,j])<-status[i]*alpha[j]+(1-status[i])*beta[j]+status[i]*re[i]*Ag

logit(pstatus[i,j])<-status[i]*alpha[j]+status[i]*re[i]*Ag+(1-status[i])*(-1000)

}

logit(p[i,5])<-status[i]*alpha[5]+(1-status[i])*beta[5]

re[i]~dnorm(0,1)

}

# Prior

prev~dbeta(0.5,0.5)

Ag~dnorm(0.0,0.1)I(0,)

alpha[1]~dnorm(0.0,0.1)

logit(s[1])<-alpha[1]

beta[1]<--1000

for (j in 2:5){

alpha[j]~dnorm(0.0,0.1)

beta[j]~dnorm(0.0,0.1)I(,1)

logit(s[j])<-alpha[j]

logit(x[j])<--beta[j]

}

se[3]<-s[3]

se[4]<-s[4]

se[5]<-s[5]

for (j in 1:2){

se[j]<-sum(pstatus[,j])/sum(status[])

}

#Estimating the accuracies of nested 56kDa PCR assay, 47kDa-based real-time PCR assay, *groEL*-based real-time PCR assay, STIC, combined ICT IgM with presence of eschar, combined *GroEL-*based real-time PCR assay with PanBio ICT IgM, and combined *GroEL-*based real-time PCR assay with presence of eschar

for (i in 1:161){

for (j in 6:12){

tdiseasetestpos[i,j]<-status[i]*y[i,j]

tnondiseasetestpos[i,j]<-(1-status[i])*y[i,j]

}

}

for (j in 6:12){

se[j]<-sum(tdiseasetestpos[,j])/sum(status[])

x[j]<-1-sum(tnondiseasetestpos[,j])/(161-sum(status[]))

}

# Prediction

for (i in 1:161){

for (k in 1:32){

for (j in 1:5){

arraymatched[i,k,j]<-equals(ypred[i,j],pattern[k,j])

}

nmatched[i,k]<-sum(arraymatched[i,k,])

matchedpattern[i,k]<-equals(nmatched[i,k],5)

}

}

for (k in 1:32){

freqpred[k]<-sum(matchedpattern[,k])

}

#Bayesian p value

for (i in 1:161){

for (k in 1:32){

for (j in 1:5){

arraymatchedobs[i,k,j]<-equals(y[i,j],pattern[k,j])

}

nmatchedobs[i,k]<-sum(arraymatchedobs[i,k,])

matchedpatternobs[i,k]<-equals(nmatchedobs[i,k],5)

}

}

for (k in 1:32){

freqobs[k]<-sum(matchedpatternobs[,k])

}

for (k in 1:32){

pvalue[k]<-step(freqpred[k]-freqobs[k])

}

pvalueSe34<-step(se[4]-se[3])

pvalueSp34<-step(x[4]-x[3])

# PPV and NPV

for (i in 1:161){

for (j in 1:12){

testpos[i,j]<-y[i,j]

testneg[i,j]<-1-y[i,j]

diseasetestpos[i,j]<-status[i]*y[i,j]

nondiseasetestneg[i,j]<-(1-status[i])*(1-y[i,j])

}

}

for (j in 1:12){

stestpos[j]<-sum(testpos[,j])

stestneg[j]<-sum(testneg[,j])

sdiseasetestpos[j]<-sum(diseasetestpos[,j])

snondiseasetestneg[j]<-sum(nondiseasetestneg[,j])

ppv[j]<- sdiseasetestpos[j]/stestpos[j]

npv[j]<- snondiseasetestneg[j]/stestneg[j]

}

}

**2.3 WinBUGS code for Model 2**

# Conditional dependence model between IFA IgM and PanBio ICT IgM

model{

for (i in 1:161){

status[i]~dbern(prev)

for (j in 1:5){

y[i,j]~dbern(p[i,j])

ypred[i,j]~dbern(p[i,j])

}

}

for (i in 1:161){

for (j in 1:2){

logit(p[i,j])<-status[i]*alpha[j]+(1-status[i])*beta[j]

}

for (j in 3:4){

logit(p[i,j])<-status[i]*alpha[j]+(1-status[i])*beta[j]+status[i]*re[i]*IgM

logit(pstatus[i,j])<-status[i]*alpha[j]+status[i]*re[i]*IgM+(1-status[i])*(-1000)

}

logit(p[i,5])<-status[i]*alpha[5]+(1-status[i])*beta[5]

re[i]~dnorm(0,1)

}

# Prior

prev~dbeta(0.5,0.5)

IgM~dnorm(0.0,0.1)I(0,)

alpha[1]~dnorm(0.0,0.1)

logit(s[1])<-alpha[1]

beta[1]<--1000

for (j in 2:5){

alpha[j]~dnorm(0.0,0.1)

beta[j]~dnorm(0.0,0.1)I(,1)

logit(s[j])<-alpha[j]

logit(x[j])<--beta[j]

}

se[1]<-s[1]

se[2]<-s[2]

se[5]<-s[5]

for (j in 3:4){

se[j]<-sum(pstatus[,j])/sum(status[])

}

#Estimating the accuracies of nested 56kDa PCR assay, 47kDa-based real-time PCR assay, groEL-based real-time PCR assay, STIC, combined ICT IgM with presence of eschar, combined *GroEL-*based real-time PCR assay with PanBio ICT IgM, and combined *GroEL-*based real-time PCR assay with presence of eschar

for (i in 1:161){

for (j in 6:12){

tdiseasetestpos[i,j]<-status[i]*y[i,j]

tnondiseasetestpos[i,j]<-(1-status[i])*y[i,j]

}

}

for (j in 6:12){

se[j]<-sum(tdiseasetestpos[,j])/sum(status[])

x[j]<-1-sum(tnondiseasetestpos[,j])/(161-sum(status[]))

}

# Prediction

for (i in 1:161){

for (k in 1:32){

for (j in 1:5){

arraymatched[i,k,j]<-equals(ypred[i,j],pattern[k,j])

}

nmatched[i,k]<-sum(arraymatched[i,k,])

matchedpattern[i,k]<-equals(nmatched[i,k],5)

}

}

for (k in 1:32){

freqpred[k]<-sum(matchedpattern[,k])

}

#Bayesian p value

for (i in 1:161){

for (k in 1:32){

for (j in 1:5){

arraymatchedobs[i,k,j]<-equals(y[i,j],pattern[k,j])

}

nmatchedobs[i,k]<-sum(arraymatchedobs[i,k,])

matchedpatternobs[i,k]<-equals(nmatchedobs[i,k],5)

}

}

for (k in 1:32){

freqobs[k]<-sum(matchedpatternobs[,k])

}

for (k in 1:32){

pvalue[k]<-step(freqpred[k]-freqobs[k])

}

pvalueSe34<-step(se[4]-se[3])

pvalueSp34<-step(x[4]-x[3])

# PPV and NPV

for (i in 1:161){

for (j in 1:12){

testpos[i,j]<-y[i,j]

testneg[i,j]<-1-y[i,j]

diseasetestpos[i,j]<-status[i]*y[i,j]

nondiseasetestneg[i,j]<-(1-status[i])*(1-y[i,j])

}

}

for (j in 1:12){

stestpos[j]<-sum(testpos[,j])

stestneg[j]<-sum(testneg[,j])

sdiseasetestpos[j]<-sum(diseasetestpos[,j])

snondiseasetestneg[j]<-sum(nondiseasetestneg[,j])

ppv[j]<- sdiseasetestpos[j]/stestpos[j]

npv[j]<- snondiseasetestneg[j]/stestneg[j]

}

}

**2.4 WinBUGS code for sceptical prior**

# Conditional dependence model between IFA IgM and PanBio ICT IgM

model{

for (i in 1:161){

status[i]~dbern(prev)

for (j in 1:5){

y[i,j]~dbern(p[i,j])

ypred[i,j]~dbern(p[i,j])

}

}

for (i in 1:161){

for (j in 1:2){

logit(p[i,j])<-status[i]*alpha[j]+(1-status[i])*beta[j]

}

for (j in 3:4){

logit(p[i,j])<-status[i]*alpha[j]+(1-status[i])*beta[j]+status[i]*re[i]*IgM

logit(pstatus[i,j])<-status[i]*alpha[j]+status[i]*re[i]*IgM+(1-status[i])*(-1000)

}

logit(p[i,5])<-status[i]*alpha[5]+(1-status[i])*beta[5]

re[i]~dnorm(0,1)

}

# Prior

prev~dbeta(0.5,0.5)

IgM~dnorm(0.0,0.1)I(0,)

alpha[1]~dnorm(0.0,1)

logit(s[1])<-alpha[1]

beta[1]<--1000

for (j in 2:5){

alpha[j]~dnorm(0.0,1)

beta[j]~dnorm(0.0,1)I(,1)

logit(s[j])<-alpha[j]

logit(x[j])<--beta[j]

}

se[1]<-s[1]

se[2]<-s[2]

se[5]<-s[5]

for (j in 3:4){

se[j]<-sum(pstatus[,j])/sum(status[])

}

#Estimating the accuracies of nested 56kDa PCR assay, 47kDa-based real-time PCR assay, groEL-based real-time PCR assay, STIC, combined ICT IgM with presence of eschar, combined *GroEL-*based real-time PCR assay with PanBio ICT IgM, and combined *GroEL-*based real-time PCR assay with presence of eschar

for (i in 1:161){

for (j in 6:12){

tdiseasetestpos[i,j]<-status[i]*y[i,j]

tnondiseasetestpos[i,j]<-(1-status[i])*y[i,j]

}

}

for (j in 6:12){

se[j]<-sum(tdiseasetestpos[,j])/sum(status[])

x[j]<-1-sum(tnondiseasetestpos[,j])/(161-sum(status[]))

}

# Prediction

for (i in 1:161){

for (k in 1:32){

for (j in 1:5){

arraymatched[i,k,j]<-equals(ypred[i,j],pattern[k,j])

}

nmatched[i,k]<-sum(arraymatched[i,k,])

matchedpattern[i,k]<-equals(nmatched[i,k],5)

}

}

for (k in 1:32){

freqpred[k]<-sum(matchedpattern[,k])

}

#Bayesian p value

for (i in 1:161){

for (k in 1:32){

for (j in 1:5){

arraymatchedobs[i,k,j]<-equals(y[i,j],pattern[k,j])

}

nmatchedobs[i,k]<-sum(arraymatchedobs[i,k,])

matchedpatternobs[i,k]<-equals(nmatchedobs[i,k],5)

}

}

for (k in 1:32){

freqobs[k]<-sum(matchedpatternobs[,k])

}

for (k in 1:32){

pvalue[k]<-step(freqpred[k]-freqobs[k])

}

pvalueSe34<-step(se[4]-se[3])

pvalueSp34<-step(x[4]-x[3])

# PPV and NPV

for (i in 1:161){

for (j in 1:12){

testpos[i,j]<-y[i,j]

testneg[i,j]<-1-y[i,j]

diseasetestpos[i,j]<-status[i]*y[i,j]

nondiseasetestneg[i,j]<-(1-status[i])*(1-y[i,j])

}

}

for (j in 1:12){

stestpos[j]<-sum(testpos[,j])

stestneg[j]<-sum(testneg[,j])

sdiseasetestpos[j]<-sum(diseasetestpos[,j])

snondiseasetestneg[j]<-sum(nondiseasetestneg[,j])

ppv[j]<- sdiseasetestpos[j]/stestpos[j]

npv[j]<- snondiseasetestneg[j]/stestneg[j]

}

}

**2.5 WinBUGS code for enthusiastic prior**

# Conditional dependence model between IFA IgM and PanBio ICT IgM

model{

for (i in 1:161){

status[i]~dbern(prev)

for (j in 1:5){

y[i,j]~dbern(p[i,j])

ypred[i,j]~dbern(p[i,j])

}

}

for (i in 1:161){

for (j in 1:2){

logit(p[i,j])<-status[i]*alpha[j]+(1-status[i])*beta[j]

}

for (j in 3:4){

logit(p[i,j])<-status[i]*alpha[j]+(1-status[i])*beta[j]+status[i]*re[i]*IgM

logit(pstatus[i,j])<-status[i]*alpha[j]+status[i]*re[i]*IgM+(1-status[i])*(-1000)

}

logit(p[i,5])<-status[i]*alpha[5]+(1-status[i])*beta[5]

re[i]~dnorm(0,1)

}

# Prior

prev~dbeta(0.5,0.5)

IgM~dnorm(0.0,0.1)I(0,)

alpha[1]~dnorm(3,0.4)

logit(s[1])<-alpha[1]

beta[1]<--1000

for (j in 2:5){

alpha[j]~dnorm(3,0.4)

beta[j]~dnorm(-3,0.4)I(,1)

logit(s[j])<-alpha[j]

logit(x[j])<--beta[j]

}

se[1]<-s[1]

se[2]<-s[2]

se[5]<-s[5]

for (j in 3:4){

se[j]<-sum(pstatus[,j])/sum(status[])

}

#Estimating the accuracies of nested 56kDa PCR assay, 47kDa-based real-time PCR assay, groEL-based real-time PCR assay, STIC, combined ICT IgM with presence of eschar, combined *GroEL-*based real-time PCR assay with PanBio ICT IgM, and combined *GroEL-*based real-time PCR assay with presence of eschar

for (i in 1:161){

for (j in 6:12){

tdiseasetestpos[i,j]<-status[i]*y[i,j]

tnondiseasetestpos[i,j]<-(1-status[i])*y[i,j]

}

}

for (j in 6:12){

se[j]<-sum(tdiseasetestpos[,j])/sum(status[])

x[j]<-1-sum(tnondiseasetestpos[,j])/(161-sum(status[]))

}

# Prediction

for (i in 1:161){

for (k in 1:32){

for (j in 1:5){

arraymatched[i,k,j]<-equals(ypred[i,j],pattern[k,j])

}

nmatched[i,k]<-sum(arraymatched[i,k,])

matchedpattern[i,k]<-equals(nmatched[i,k],5)

}

}

for (k in 1:32){

freqpred[k]<-sum(matchedpattern[,k])

}

#Bayesian p value

for (i in 1:161){

for (k in 1:32){

for (j in 1:5){

arraymatchedobs[i,k,j]<-equals(y[i,j],pattern[k,j])

}

nmatchedobs[i,k]<-sum(arraymatchedobs[i,k,])

matchedpatternobs[i,k]<-equals(nmatchedobs[i,k],5)

}

}

for (k in 1:32){

freqobs[k]<-sum(matchedpatternobs[,k])

}

for (k in 1:32){

pvalue[k]<-step(freqpred[k]-freqobs[k])

}

pvalueSe34<-step(se[4]-se[3])

pvalueSp34<-step(x[4]-x[3])

# PPV and NPV

for (i in 1:161){

for (j in 1:12){

testpos[i,j]<-y[i,j]

testneg[i,j]<-1-y[i,j]

diseasetestpos[i,j]<-status[i]*y[i,j]

nondiseasetestneg[i,j]<-(1-status[i])*(1-y[i,j])

}

}

for (j in 1:12){

stestpos[j]<-sum(testpos[,j])

stestneg[j]<-sum(testneg[,j])

sdiseasetestpos[j]<-sum(diseasetestpos[,j])

snondiseasetestneg[j]<-sum(nondiseasetestneg[,j])

ppv[j]<- sdiseasetestpos[j]/stestpos[j]

npv[j]<- snondiseasetestneg[j]/stestneg[j]

}

}
